# The challenge of sarcomas: the patient advocacy group perspective

**DOI:** 10.1186/s13569-019-0121-6

**Published:** 2019-07-17

**Authors:** Roger Wilson

**Affiliations:** Sarcoma Patients EuroNet e.V./Association, Untergasse 36, 61200 Wolfersheim, Germany

**Keywords:** Sarcoma, Patient advocacy, Multi-Disciplinary Management, Quality of life, Cross-border treatment

## Abstract

**Background:**

The patient advocacy agenda covers every aspect of cancer treatment and care. This inevitably means that this review covers almost everything that patient advocates are involved with, whether locally, nationally or across national borders. Over more than 15 years of working as an advocate I have been involved in representation and/or discussion about almost all the topics which follow.

**Structure of the review:**

I have broken this review into five main sections and have identified within each an advocacy priority. This is then supported by a number of further areas of advocacy activity. The review starts with a look at what advocacy is and closes with a short discussion on how sarcoma advocacy is structured internationally. The five sections are: (i) Clinical challenges, (ii) Challenges for healthcare systems, (iii) The cross-border challenges, (iv) Keeping up to date, (v) Research. The five priority challenges, one within each area above, are listed respectively in order with the above: (i) Earlier accurate diagnosis and primary treatment, (ii) Multi-Disciplinary Management, (iii) Cancer registration and patient data, (iv) Quality of life and PROs, (v) Patient involvement.

**Conclusions:**

Across many of the challenges which I identify good progress is being made. The importance of the partnership with the professional specialists in treating sarcoma cannot be emphasised too strongly and the leadership of key people, whether patient advocates or professionals, is acknowledged. There are challenges indicated which have yet to be properly addressed. Inevitably some of them have characteristics which make them especially problematic and they tend to drop lower on everyone’s agendas. This does not mean we should forget them.

## Background

The patient advocacy agenda covers every aspect of cancer treatment and care. This inevitably means this review covers almost everything that patient advocates are involved with, whether locally, nationally or across national borders. Over more than 15 years of working as an advocate I have been involved in representation and/or discussion about almost all the topics which follow.

### Patient advocacy groups

Not all patients are advocates and not all patient advocates are patients. It may seem a strange comment to make but it is a truism which should be remembered. Patient advocacy in cancer began during the 1990s with the breast cancer community recognising what was being achieved by HIV/AIDS patients, in particular their involvement in research and the ways they were able to campaign for clinical trial results to move into standard clinical practice rapidly.

In the sarcoma patient community the formative years for advocacy were the late 1990s. In the USA patients had come together through email lists, and the enthusiasm of two patients had created the Sarcoma Alliance on the West Coast, as the first patient organisation specifically for sarcoma. The emergence of imatinib as a treatment for GIST, previously with no effective treatment once recurrent, started a movement of change. In the USA two patient groups formed with a patient-led focus on GIST—the Liferaft Group and GIST Support International (GSI). In Europe fledgling groups emerged which were brought together with the US groups by Novartis in the New Horizons programme, to focus on GIST and imatinib. New Horizons was moderated and chaired by Kathy Redmond, who in an article in BJUI in 2003 defined patient advocacy thus:

*Patient organizations represent the interests of a defined group of patients, and can be identified because they have patients as members and by the presence of patient representatives on their boards. Most patient organizations are not involved in advocacy; they focus their efforts on providing support and information. In contrast, patients’ advocates bring their influence to bear at all levels of health policy decision-making, with the aim of improving access to high-quality treatment and care, and ensuring that patients’ rights are upheld. Patient advocates influence the political agenda by raising public awareness of inequities and problems confronted by patients, and by seeking representation on key committees. They also lobby politicians and other policy-makers directly to ensure that patients’ viewpoints are considered when policy decisions are made.*

The oldest European sarcoma and GIST patient organisation is Sarcoma UK, founded in 2003, although Das Lebenshaus in Germany and Info Sarcomes in France quickly evolved to support sarcoma alongside their GIST support work. In Poland the other early starter was Stow Pomocy Chorym Na Miesaki Sarcoma. GIST Support UK span out of Sarcoma UK in 2008 and in 2009 all these groups plus GIST groups from Italy, Poland, Romania and Switzerland were encouraged by the EU funded Conticanet project, led by Professor Jean-Yves Blay, to come together to form Sarcoma Patients Euronet (SPAEN).

The distinctions which Kathy Redmond makes in her definition quoted above are important to recognise. The national groups which are members of SPAEN are primarily groups providing emotional and social support, developing the resources which allow them to advise and inform patients who contact them. Most of them also undertake some level of advocacy work in support of their wider objectives (e.g. campaigning for earlier diagnosis), to help specific groups of sarcoma patients (e.g. gaining access to a new treatment) or individual patients facing exceptional challenges. Most of them also support research into sarcoma in some form, helping get studies underway and encouraging recruitment or, as happens in the UK, providing funding for research. The three UK members of SPAEN committed over £1m to research in both 2017 and 2018—small enough when the whole need is considered but sums which were unthinkable 10 years ago.

SPAEN has evolved over the years to a membership of more than 40 groups from 23 countries [1]. The reach is now beyond Europe to Israel, India, Kenya, Australia and the USA. There is a strong similarity in the objectives of all these groups, starting with information, advice and support, extending to involvement with their treatment specialists in a variety of activities including research, and to advocacy and political lobbying.

### The patient/professional partnership

One of the characteristics of the sarcoma community has been the development of active partnerships between patients and the specialist doctors who treat sarcoma patients. This is evident at an international level as well as at a national level in those countries where a network of specialist treatment centres is in place or is developing.

No-one denies that doctors have a valuable role as advocates for their patients, especially when obtaining a specific treatment which lies outside standard care but at the same time no one will deny that there are aspects of the wider advocacy need where patients bring a particular impact. It is in coming together as partners, balancing the available skills and experience to find agreement on the nature of the change being addressed, where the relationship between patients and professionals can take on particular effectiveness. This can be in clinical areas, research, service development, in issues of treatment funding or establishing regional or national policy.

Examples exist of partnership in advocacy action in all these areas in a number of countries and it is noticeable that the countries which have robust and established patient/professional relationships seem to be developing the more effective treatment networks. It does however take time. It has been likened by one advocate to “climbing a cliff: you move one limb at a time, you don’t look up and you don’t look down, you just keep climbing.” (Quote from conference presentation).

Leadership is a major factor here, both from professional groups and patient groups. Sharing concerns, understanding the gaps, identifying opportunities, working together to lobby decision-makers and influencers, also helps build the understanding. Where leaders from both sides have a good personal relationship the effectiveness of the partnership increases.

### The challenge of sarcomas

#### Overview: the sections and key priority areas

##### Section 1: Clinical

Talking to patients on a regular basis over several years it is easy to identify medical and clinical issues which are current for many patient groups, whether they are actively working as advocacy groups or simply providing support, information and advice to their patients. These problems are well understood within the sarcoma community although the ability to address some of them is limited and healthcare systems need to pay more attention to these challenges too.

- The need for earlier accurate diagnosis
  - A need for specialist pathology
  - Access to specialist surgery
- Rehabilitation and age discrimination especially considering the younger age range of sarcoma patients
- Recurrence and development of advanced disease
- Palliative care

Addressing these challenges also involves a need for research.

##### Section 2: Healthcare systems

There are also healthcare system challenges for sarcoma. The situations in different countries vary hugely, with organised national networks in some countries, a developing specialist network in some, and little recognition of specialist requirements in others.

- Recognition of the need for Multi-Disciplinary Team (MDT) management of sarcoma treatment
  - Development and adoption of treatment guidelines
- Access (including funding) to specialist treatment for exceptional and rare needs (e.g. proton beam for pelvic Ewings sarcoma)
- Second opinions
- Facilitation of patient referral into specialist care
- Recognition of the value of specialist nurses, physiotherapists etc.
- Quality of information available to patients

Once again there are research needs which would help address some of these challenges.

##### Section 3: Cross border

There are cross-border challenges, where countries must work together. This includes the recognition by major organisations such as the European Union of rarer cancer challenges which include sarcoma but do not focus exclusively on sarcoma. The contribution of the sarcoma community to the rarer cancer challenge is significant. Challenges include:

- Cancer registration
  - Patient data
- Cross border treatment—Reference Networks, EURACAN
- Cross border research—science should know no borders
- Drug regulation
- Innovative clinical trial methodology
- Medical education and training

This topic takes on a particular poignancy with the impending departure of the UK from the European Union. The UK has been a leader and a beneficiary in EU funded research and cross-border alliances.

##### Section 4: Keeping up to date

Other patient advocacy priorities stem from the need to keep up to date with what is happening in other areas of cancer research and treatment. Sarcoma is a cancer and the evolution of new themes in cancer treatment and care are relevant.

- Quality of life and PROs—towards holistic care
- Precision medicine—Next Generation Sequencing (NGS)
- Survival—“treatment is a process, survival is a black hole” (quote from patient)
- Tissue banking and access to samples
- Follow-up

Yet again the need for research is intertwined with these challenges.

##### Section 5: Research

Clinical practice and research prove inseparable during this review but for completeness we should look at two important challenges in research separately.

- Patient involvement
- A question

##### Summary

It may seem that the patient advocate’s shopping list is rather broad. However, that is the nature of patient advocacy. The individual may focus on a small subset of these challenges but the whole spectrum is relevant. I have identified one of the issues in each section as a priority challenge and address each of the others in a shorter paragraph to explain the challenge it presents.

## Discussion

### Section 1—Clinical challenges

#### The priority challenge—earlier, accurate diagnosis and primary treatment

##### Earlier diagnosis

Sarcoma is a rare disease which manifests itself in a multitude of different ways affecting almost any part of the body. Even if suspected it is hard to confirm. If a proper pathway is followed, referral to a specialist MDT, or surgeon, should result. What comes through from research is that earlier diagnosis will mean tumours that are smaller and it is known that tumour size is a prognostic indicator.

However, the rarity of sarcoma means that any primary care doctor in the community will see few cases in a whole career and these cases will most probably be dissimilar, even if they affect the same part of the body. What is less readily recognised is that doctors in secondary care, local hospitals, are also unfamiliar with sarcoma. There may be a greater likelihood of suspicion than in primary care, although this can lead to an incomplete or inaccurate diagnosis and an inappropriate excision. Data on this situation are almost impossible to find. Presentations at a recent SPAEN Conference from healthcare systems which gather data gave us an idea of the probable scale of the problem. We believe that in many countries when this happens it is not recorded or talked about.

France has a strong network of specialist surgical centres, Netsarc. It has 26 centres evenly spread across the nation. Their data reported to SPAEN indicates that 37% of patients are referred to a Netsarc centre prior to surgery while 55% have surgery outside the network. Of this latter group 77% have a local recurrence—which is generally recognised as a poor prognostic indicator.

In the UK England has a network of 14 treatment centres, all treating soft tissue sarcoma. Five of them are funded to treat bone sarcomas as well. Paediatric cases are treated in specialist paediatric cancer units. In the centralised NHS doctors are mandated on suspicion of sarcoma to refer to a specialist centre. However, similar to France over half of soft tissue patients are first diagnosed outside the network although 55% have their first surgery in a specialist centre. The percentage for bone sarcoma is 80% having surgery in a specialist unit. Survival at 12 months differs—3% positive difference between those referred and those not referred prior to surgery for bone and 5% difference for soft tissue sarcomas. That alone supports the argument for earlier, and correct, diagnosis.

In the Netherlands the incidence of sarcoma is similar to that of the UK. Registration covers all cases. Even though there is a well established network of seven centres specialising in sarcoma only one-third of patients are seen by them in the first instance. In recent years 20 other hospitals have treated up to four patients a year, rather than referring following a locally confirmed diagnosis.

Given the effort and investment which has been made by these three healthcare systems over the last 10–12 years these figures are disappointing. One of the penalties of improving the referral network is evident from England where all the specialist units have seen the numbers of patients with benign tumours referred for specialist diagnosis on suspicion increase dramatically. This is happening despite, or perhaps caused by, a growth in diagnostic ultra-sound services in the community. Specialist units have introduced steps such as nurse-led triage to handle the burden of inappropriate referral by primary care doctors—a professional irony which should not be lost.

Drawing a conclusion from the data from the UK and France is quite straightforward. These are two sophisticated, although very different, healthcare systems. They have good networks of specialist pathologists, even if a greater number of them would help. We have no evidence to suggest that any country is immune from this diagnostic problem. Further data are needed here. We think that something quite radical is needed to address the issues of getting an earlier correct diagnosis. This needs to be applicable to every healthcare system and requires some creative thinking.

##### Accurate diagnosis

If earlier diagnosis is important accurate diagnosis is equally critical. The relationship between pathology and treatment is often not well understood by patients. There is an assumption that science can be left to those who have been trained and they can be trusted to get it right. The complexities of sarcoma pathology and the understandings that a histopathologist has to acquire in order to become a sarcoma expert remain a mystery until explained. Sometimes it seems they are also a mystery to pathologists who rarely see a sarcoma and have few of the required understandings.

Almost without exception the reports of specialist pathologists given to SPAEN suggest that 30–50% of initial diagnoses made outside a sarcoma expert network are incorrect. Often it is a matter of failing to identify the correct histotype among the many which make up the spectrum of sarcoma. Sometimes it is a failure to identify a benign tumour correctly, or more worryingly a benign tumour is referred for an opinion, which the specialist then identifies as malignant.

The impact of an incorrect pathology report can be substantial. Different sarcomas grow in different ways, some have a pseudo-capsule, others are infiltrative. This affects the approach to surgery. Tumour size may affect neo-adjuvant radiotherapy decisions. Tumour grade may affect the approach to adjuvant therapy. Some tumours are more indolent than others, affecting follow-up decisions. There are many other situations affected by the pathology so seeking an accurate diagnosis is every bit as important as an early diagnosis.

##### Primary treatment

The adjunct to an earlier and accurate diagnosis is the first curative treatment, usually surgery, with the aim of delivering the maximum benefit, based on the diagnosis. The issue of access to specialist surgery is not confined to the relatively simple case of receiving and acting on a referral from primary (or secondary) care at a specialist (tertiary) centre or MDT. Ensuring the appropriate surgical expertise for each individual patient is a further issue.

There are many sarcoma cases where the surgery is relatively straightforward. Appropriate imaging acts as a guide to decisions about surgical margins, limb preservation, reconstructive surgery etc. However there are groups of sarcoma patients where additional expertise is required. Head and neck sarcomas need a surgeon with appropriate experience in dealing with these delicate structures, some paediatric limb tumours need additional orthopaedic expertise (e.g. endoprosthetics), pelvic and spinal tumours present challenges, and within the retroperitoneal and abdominal spaces it is now recognised that surgery by a sarcoma specialist with the right experience offers patients the best chances of a cure. It is also where far too often patients do not receive it.

There are many surgeons who undertake surgery within the abdomen. Gastrointestinal surgery is common for cancer and other GI problems. This does not however mean that a good GI surgeon is best equipped to undertake surgery on retroperitoneal sarcomas. These RPS tumours can grow very large and because they do not directly affect the functioning of an organ they give few symptoms and can go disregarded for months and years. Eventually they reach a size where they interfere with a nerve or put pressure on an organ and investigations start. As they grow RPS tumours can encase organs such as kidney or spleen, and these are then threatened by the necessary surgery. The Transatlantic & Australasian RPS Working Group (TARPSWG), an international consensus group of experienced surgeons, has developed guidance and these should be observed [2, 3].

We hear of too many cases where guidelines are not followed. Localised recurrence for RPS is common. Further radical surgery may not be possible and experience shows that it is unlikely to prevent later unresectable recurrence or distant metastasis.

#### Further challenges

##### Rehabilitation

The range of rehabilitative care varies country by country and is frequently offered through local providers where there is little or no familiarity with the particular needs of sarcoma patients. Healthcare systems generally have poor recognition of cancer patients below the age of 50, which accounts for some 35% of all sarcoma cases. The range of requirements can be extensive. Maintaining fertility has to be addressed prior to primary surgery and adjuvant therapy, while orthotics/prosthetics have long term cost implications, and supporting return to work means addressing attitudinal issues within the employment market. This is particularly important to younger patients and there are reports of discrimination on the grounds of having been treated for cancer.

A systematic review undertaken in the UK in 2012 revealed only 3 studies in sarcoma rehabilitation, one for Kaposi’s sarcoma. Widening the search criteria brought up a further three, all looking at issues of amputation [4].

Social re-integration is a rarely described need for older cancer patients but with the young adult group in particular is a need which should not be ignored. Families can be a powerful aide, which simply highlights the issue for those who do not have that kind of support.

Rehabilitation is under-researched, especially among the younger age groups. Some patients have extensive needs which community care cannot always address. This overlaps with the wider issues usually identified as “survivorship”, where a positive research-led approach has been climbing the strategic agenda in cancer in recent years.

##### Advanced disease

Considering the needs of patients with advanced disease, and addressing their expectations, requires a growing intelligence network and an agile understanding of what each new therapy means for patients if a patient group is to be effective. Providing advice in an age when new treatments are proliferating also calls for new kinds of evidence which can be used to support patient choices and decision-making. The quality-of-life agenda across cancer is now well identified.

New agents come into trials and then, hopefully, into standard clinical care. There are fewer of them in sarcoma than in most of the more common cancers but they are slowly coming.

There has been a growth of ablative therapies capable of treating locally recurrent and early metastatic (oligo-metastatic) tumours. There is little published research on effectiveness. Techniques in surgery are also moving forward. The days when single agent doxorubicin was the first-line treatment for almost every patient with advanced sarcoma looked to be over when oloratumab was licensed in 2017 following positive data from a Phase 2 trial. However the Phase 3 study showed no benefits and the drug was withdrawn. Intriguingly the response to doxorubicin + placebo (the control arm of the Phase 3 study) was the best yet seen for doxorubicin in sarcoma research. Oloratumab may re-surface, targeted at specific sarcomas. We have yet to hear more.

There is no easily defined or universally available algorithm to support clinical recommendations and, by implication, to help patient advocacy groups understand decision-making when metastatic sarcoma is diagnosed. The NCCN and ESMO guidance seem to be interpreted as much by the therapies that are most favoured locally as by any other factor. It is an area where anecdotal evidence proliferates and the shortage of good prospective studies, which are difficult to design, is noticeable. Also noticeable is the absence of retrospective research, especially good quality multi-site series covering metastatic patients. The absence of structured quality of life evidence, which has been aggregated and allows comparison, does not help either.

Subjective outcomes (Patient Reported Outcomes—PROs) are an appropriate and valuable route forward. Given the prognostic nature of advanced disease PROs should be co-primary endpoints in all studies. The reluctance of some research clinicians to support this step may reflect the poor standard of some of the available tools, or the shortage of skills in analysing and interpreting the data gathered.

##### Palliative care

A patient can become committed to the idea of a cure even though the clinicians treating them know that this is unrealistic. Advocates can buy-into this falsehood and lose sight of an important perspective. At some point in the advanced disease pathway a patient will either respond to a clinician’s question, or make up their own mind, that no further ‘curative’ treatment is appropriate for them, accepting symptomatic palliative care as the right choice. Good quality of life data may influence more patients to make this choice earlier, avoiding treatment with toxic drugs close to the end-of-life which will have no effect other than deteriorating the quality of life. We have no evidence in sarcoma to support the possibility, opened up by the Temel study [5] in lung cancer, that proactive palliative care can enhance and even extend life. This hypothesis needs to be explored.

### Section 2—Challenges for healthcare systems

#### The priority challenge—Multi-Disciplinary Management

##### The Multi-Disciplinary Team (MDT)

It is generally accepted that the Multi-Disciplinary Management of patient treatment and care leads to more positive overall outcomes. It certainly adds to patient satisfaction as the transition from one specialist doctor to another is smoother, usually managed by a specialist nurse or dedicated co-ordinator who remains the key point of contact covering all eventualities. The individual members of a sarcoma Multi-Disciplinary Team (MDT) may have other clinical specialties but they should all have dedicated sarcoma time, which must include the sarcoma MDT regular meetings to review current patients.

This indicates that the MDT should include all the relevant specialists which could treat a sarcoma. The time they dedicate to sarcoma will be dictated by the workload that emerges. The key full-time team member should be the specialist nurse.

There is a strong wish among patient advocates to have some method for certifying MDTs but ideas about what criteria should apply are varied and the proposed methods of applying them are in many cases impracticable. Having such an ‘approved’ list also raises the challenge of how to remove an approved centre from the list when the criteria are no longer met. The EURACAN ERN may go some way towards addressing this need but it is early days.

In a healthcare system where no specialist MDTs exist the need for them can be hard for patient advocates to articulate persuasively without sounding over critical of the systems which are in place already. Even if that criticism may be justified. The SPAEN Policy Paper [6] on ‘Quality Care in Sarcomas’ puts forward a pathway of care which can only be effectively managed within a multi-disciplinary environment. This Policy Paper is a tool which we hope that healthcare systems without MDTs specialising in treating sarcoma, can use positively.

##### Treatment guidelines

An MDT needs to have protocols to guide its work. These should, wherever possible be supported by evidence and evidence-based consensus guidelines. In a heterogonous group of diseases like sarcoma there are situations when extremely rare cases are diagnosed. Therefore guidance should be advisory, not mandatory. Both ESMO in Europe and NCCN in the USA offer a comprehensive look at treatment, while leaving open those very exceptional circumstances and allowing for the exceptional patient when a different approach is appropriate. In the UK the British Sarcoma Group has its own guidance, which closely follows the ESMO approach, but which is framed in such a way that the UK’s unique healthcare commissioning and funding structure can adopt it en bloc. One noticeable feature is that such guidance is developed by a consensus group of scientists and doctors, usually without patient involvement.

#### Further challenges

##### Exceptional and rare needs

As indicated above there are exceptions and instances where reference to individual expertise is valuable, or even necessary, to support treatment recommendations. In these cases, which lie outside usual guidance, it is not unusual for specific funding to be required.

Some of these situations occur early in treatment. The use of radiotherapy to treat Ewings sarcoma is well established but traditional approaches can create morbidity. This is undesirable in younger people because the disease is often curable. The use of proton beam radiation is now well established but facilities are costly to build and treatment is not automatically funded in many healthcare systems. Funders need to have fast-track authorisation procedures which can respond to such situations.

This becomes more challenging when disease becomes advanced and systemic drug treatments in a palliative setting are proposed. A significant proportion of sarcomas do not respond to standard treatment when metastatic disease appears. For some chemotherapy shows no value and whatever mutations are found in genetic sequencing have no treatments available to address them. This is an area where the need for new drugs and innovative approaches to treatment stands out—but someone has to fund them. There is certainly a role for advocacy here which requires both clinicians and patients working together. A SPAEN/EORTC Roundtable consensus event in July 2018 is a step on this difficult advocacy journey.

##### Second opinions

It is probable that more second opinions are sought in rarer cancers than in the more common ones but by definition those able to offer them are fewer and more widely scattered. With sarcoma, where 70+ pathological diagnoses are possible and the ability to distinguish between different histological sub-types is itself scarce, it is likely that there will be many more pathology referrals. Funding this process is an important part of a healthcare provider’s role, without it patient outcomes will be poorer. We know of instances where a busy second opinion workload in pathology is not funded. Healthcare providers seem to want to rely on assurances of professional expertise rather than being ready to accept mis-diagnosis data as evidence of the value of scarce specialism.

In the same way decisions made by single clinicians, especially when stepping outside the boundary of their primary discipline (e.g. a surgeon recommending chemotherapy), should be considered by a specialist. This would happen automatically within an MDT, a further argument for adopting that structure. Advocacy advice in this circumstance is: if in doubt seek a second opinion from someone with recognised expertise.

Similarly in extremely rare cases seeking the advice of those who have experience is important. We have seen the situation arise where a doctor has sought the advice of a patient advocacy group because their knowledge of the spectrum of patient experience could identify who had treated a certain condition most recently.

##### Patient referral

A traditional medical system relies on patients being diagnosed, then treated or referred if appropriate to specialist doctors who can treat them, with further onward referral over time as necessary. One of the objectives of the MDT is to reduce this need, avoid patients being lost in the system, eliminate uncertainty and to concentrate in one hospital, or group of neighbouring hospitals, all the expertise needed to treat a patient group. Where a more traditional referral system exists and specialists are not widely recognised there is an additional factor. This is when doctors, or their hospitals, are financially rewarded for providing diagnostic services or treatment which proves unnecessary or inappropriate. In most healthcare system in Western Europe this is not an issue but in some parts of the world it does appear to be happening, or give the impression that it is happening.

Patient advocates do their best to encourage referral to appropriate expertise. While healthcare systems are not prepared to penalise hospitals which act inappropriately this is an advocacy role which will continue.

##### Specialist nurses, physiotherapists etc

A few healthcare systems or hospitals, notably in the UK and Ireland, recognise the value brought by nurses and other healthcare professionals when they are allowed to specialise in the treatment and care of a specific group of patients. The benefit is twofold—both physical and psychological, the latter being under-recognised and largely unresearched.

Clinical Nurse Specialists in Ireland and the UK (known as CNS) interact with patients in a different way from doctors, even when they are working alongside them. This is intuitively recognised by patients and anecdotally appears to apply even when the usual gender balance is reversed, the nurse being male and the doctor female. Where a CNS is available their value is as autonomous practitioners who are central to the patient journey, raise the quality of care, listen, advise and organise. They can be powerful advocates on behalf of their patients. They do not replace doctors but they can develop knowledge and skills which enable them to deliver some of the expertise which doctors are usually called on to provide [7].

The relationship between patient advocates and CNS is usually strong. They share similar objectives and have complementary experience and skills.

##### Quality of patient information

Healthcare systems have never engaged properly with providing patient information of a high standard, accessible where and when it is needed, and always up to date. The so-called information age has created a proliferation of opportunities to publish health information from amateur, sometimes dangerous sources, on the internet. Patient advocacy groups have to develop multiple media versions of their information to ensure that up to date and reliable information is available.

Providing a reliable and authoritative source of information about sarcoma which can be made available in any language was one of the ambitions expressed when Sarcoma Patients Euronet started. The website, re-launched in summer 2017, is realising that ambition. In March 2018 there were 10 languages covering the major parts of the website. Several member associations have also adopted the wording from the site, which had been thoroughly checked by clinical experts, for reproduction on their own organisation websites. This has been advocacy in action.

### Section 3—The cross-border challenges

#### The priority challenge—registration and patient data

##### Registration

Cancer registries are one of the hot topics in the field of cancer. A properly designed and maintained registry, which has all the data relevant to patients, should be a source of research for epidemiologists, clinicians, and methodologists with the aim of improving healthcare.

We have some valuable experience with registries from the UK to illustrate the challenges that exist. In 2008 steps began to integrate hospital data, cancer registry data and national registration data (especially deaths). Later addition of other datasets has included radiotherapy and chemotherapy data. Cancer registration in the UK is mandatory for NHS hospitals. When registration data from eight regional registries was merged it was soon found that a lot of hospital data on sarcoma did not show up. There was a huge mismatch in the numbers of cases. It emerged that each of the regional cancer registries approached coding sarcoma in a slightly different way, and even then some hospitals entering data did not conform. Establishing conformity, correcting historical data, creating data quality all took time. Nationally the number of sarcoma cases increased well in excess of the numbers expected even by a growing population—from about 1800 cases reported in 2002 in England to 3800 cases reported in 2010 and over 5000 once all UK data was included. A lot of the problems were with visceral sarcomas being registered as a cancer of the affected organ. The UK incidence for sarcoma which has resulted is > 75 cases per million of population including all sub-types of sarcoma. It is greater than numbers from Scandinavia and the Netherlands.

There are no data to indicate whether other national registries face the same underlying problems that the UK has had to overcome but it is likely that many do. Data from the American Cancer Society [8] indicates the potential scale of the issue. They forecast 16,490 cases of sarcoma in the USA in 2018 (13.040 STS, 3450 bone). Applying the 75 pm incidence to the US population suggests a total of over 24,000 cases, a figure 50% higher than the ACS forecast. It can be expected that different populations may have a different incidence but is a difference this large probable?

Research is needed to uncover the true numbers of sarcoma patients and it can be anticipated this could also reveal issues about how sarcoma is diagnosed and treated, as it has done in the UK.

##### Patient data

Accurate registration data will enable accurate analysis of that data and provide a base on which a wider patient profile can be built. This is one of the fundamental ideas of the ‘big data’ revolution which the information technology industry is so keen to get to grips with in cancer. It also lies behind the potential seen for artificial intelligence to support improvements in care.

There are indications of the real value which this approach can bring. As the UK data was being collated and corrected a gap appeared indicating that there were issues with the diagnostic pathway which patients were following. Analysis showed that many more patients than anticipated were first presenting at A&E. These tended to be patients with more advanced incurable disease, many of whom were never treated by an MDT. They returned home to community based palliative care and were never included in the cancer registry although sarcoma was noted as a cause of death in the national registry [9]. This analysis, covering all cancers, has led to the introduction in the UK of a new oncology discipline associated with acute presentation of cancer. For sarcoma it has also led to better community based imaging and new guidance for general practitioners.

So-called ‘big data’ is, however, more than just cancer registry and clinical data. It should include ‘quality of life’ data, or data gathered from PROMs (Patient Reported Outcome Measures). This way we would be able to see the true value of a treatment from both observed and experienced viewpoints, rather than the blunt, sometimes simplistic, objective views we have at the moment. Extension of PRO data capture into routine clinical care would be made easier by smartphone apps and could offer large amounts of valuable information very quickly, given appropriate access, data security and analytical tools.

Neither doctors nor patient advocates have yet got to grips with the potential that information technology could bring and we need to start thinking constructively, and talking together with those who might bring expertise and investment.

#### Further challenges

##### Cross border treatment—reference networks

For rare cancers cross-border treatment seems an ideal approach to addressing the scarcity of true expertise. The European Union has no say over national healthcare systems and the funding of cross-border treatment carries uncertainties. The introduction of EURACAN, the rare cancers reference network, through the EU initiative on rare diseases, will hopefully make things easier and provide some answers. If nothing else the development of a Reference Network which provides structures for cross-border referral and second opinions will uncover some of the underlying socio-political and financial issues which are unresolved and need addressing.

EURACAN is an important step along the difficult road of bringing all of Europe’s sarcoma patients within reach of specialist treatment and care.

##### Cross border research

‘Science should know no borders’ is a mantra being severely challenged by the Brexit process through which the UK is leaving the European Union. Scientists have known no borders within Europe until now. One of the hidden immediate outcomes from the UK’s decision has been that visiting professorships and scientific fellowships, usually a 5 year appointment, are not being taken up by American, Canadian and Australasian scientists because they can not be guaranteed access to the best European students and post-doctoral researchers.

It is not my intention to get into politics here but it would be regrettable if sarcoma research, already challenged by needing to build international projects, found obstructions put in its way through new barriers hindering, or even preventing, people working together or accessing funding sources.

##### Drug regulation

It is hard to recall the time before EMA took over drug regulation in Europe and it is therefore with some relief that I noted that, with Brexit on its agenda, the UK government has stated its wish to remain a partner within EMA although this may not prove realisable. The challenge for patients which focuses on EMA is that of ensuring that the patient experience is fully taken into account when new drugs are appraised for marketing approval. It is not EMA’s job to tell manufacturers how to do their work, they can only tell the manufacturer what they want to consider and how they want that data presented. The EMA process is extremely rigorous and we can be assured that there are no compromises on safety. However quality of life data has been low on the agenda in the past and is only now starting to rise up it.

The lack of good patient reported outcome (PRO) data in sarcoma studies in the past is noticeable. Imatinib, sunitinib, trabectedin and mifamurtide (all approved more than 10 years ago) had none at all. Pazopanib (approved in 2010) had some useful QoL data, the Phase 3 study of oloratumab in 2017/18 had PROs as secondary outcomes, but most of the other targeted agents trialled in sarcoma more recently have nothing significant.

We currently lack sarcoma specific PRO instruments which will give us a clearer picture of the value to patients of a new agent. The objective medical data is not enough on its own and a comprehensive and consistent approach, understood by all researchers working on sarcoma, would be a good first step.

##### Innovative clinical trial methodology

The Rare Cancers Europe initiative led by ESMO has focussed on clinical trials and is working with EMA and industry on implementing new ideas, including innovative designs, probability statistics, meaningful surrogate endpoints etc. for rarer cancers including sarcoma. The heterogeneity of sarcoma means that as sub-types become better understood through genetic sequencing new treatment targets appear but they cover smaller and smaller groups of patients. The methods by which small Phase II studies can become registration studies are now established and this work continues with discussion about patient groups too small for even a Phase II study.

Patient advocates support ESMO and the Rare Cancers Europe working group in this work and EMA for taking seriously the challenge of using early phase studies in regulatory decision-making.

##### Medical education

Doctors in training rarely spend any time studying sarcoma. When questioned young doctors have said it amounted to one half-day of lectures in a 4 year course and any experience during clinical training was down to chance. Within the overall contexts of healthcare and medical knowledge which need to be communicated we can understand this. Doctors who wish to specialise in treating sarcoma need to train within a specialist MDT and to experience fellowships with experts in other centres, perhaps in foreign countries. There are also occasional cross-border training opportunities such as the ESurge Masterclass on retroperitoneal surgery conducted in 2016 in Paris [10].

Our professional partners are acutely aware of the challenges of acquiring and building expertise and we, as patient advocates, support them in addressing this challenge.

### Section 4—Keeping up to date

#### The priority challenge—patient reported outcomes

##### Quality of life and PROs

There have been many studies pointing to the inadequacy of quality of life appraisal in cancer clinical research. Sarcoma is not exempt from these criticisms, although most drug studies until recently have not considered quality of life at all so they escape the criticism of poor methods, bias and poor data quality. As far back as 2006 EMA [11] suggested that QoL endpoints could offer co-primary outcomes in cancer research but it seems that no-one has taken up that challenge.

Reference to quality of life appraisals has been made several times already in this review. It is not our place here to discuss the strengths and weakness of what is available, it is enough to state that actions are being taken to create more accessible and effective tools, standards and guidance [12].

A study in advanced sarcoma looking solely at quality of life is being discussed in the UK and this would give valuable data to clinicians and patients facing the difficult treatment decisions at this stage in the disease. There have been plans to develop a sarcoma specific adjunct to the EORTC QLQC-30 tool, although the challenges of handling the wide range of tumour location and histology have not yet been resolved. At Radboud University hospital in Nijmegen the Profiles Registry recording quality of life is a core component of all cancer care [13], with patient reported measures informing individual care. It is also being introduced at the Royal Marsden Hospital in London. A sarcoma specific tool is in development at University College London [14] while at the Christie Hospital in Manchester there has been a project, Plan Be, taking a holistic view on rehabilitation and care following treatment with a focus on patient choice and quality of life. This kind of work needs to become mainstream. The Desmoid group within the sarcoma community is showing the way, planning the use of PROs in a forthcoming study.

QoL tools and Patient Reported Outcome Measures (PROMS) should be integrated into clinical research but perhaps more importantly developed to enable a longitudinal approach so that we can appraise the whole pathway of treatment and care. It is possible that QoL will travel this road very easily as overall standards in cancer research and care are evolving. Sarcoma could lead the way.

#### Further challenges

##### Precision medicine—new generation sequencing (NGS)

Some of the leading research in sarcoma is also leading clinical cancer research. The study of BLU-285 (avapritinib) in GIST, with a particular target for those patients with a D842V mutation in PDGFRa, has been one of the leading studies relying on new methods for genetic analysis. The approval or larotrectinib by FDA in 2018 for cancers expressing the NTRK gene, some of which are sarcomas, showed that the regulators will respond to this kind of precision outside traditional histologically defined tumour boundaries, although the costs of treatment with such targeted therapies is high.

This move towards greater ‘precision’ in medicine is a very welcome development. It is a response to the identification of gaps in care as the sophistication of new treatments increases. The issues of funding and regulation, which we discussed earlier, clearly have an impact on how treatments of this kind can move into standard clinical practice when proven effective. The challenge is that proving them effective without the ‘gold standard’ of the Phase 3 RCT is problematic. The over riding principle is that response to a targeted therapy by patients which have that target in their tumour creates the need for a system for quick regulatory approval and for systematic patient selection. We need to add to that ongoing data-gathering to provide the ‘real world evidence’ which can go some way to providing the certainties previously offered by a randomised trial.

The UK’s 100,000 Genome project, funded by the government’s Department of Health, is focussing on sarcoma as one of its rare cancer priorities. The aim is to be able to match patients with genomic mutations to treatments for those mutations. Rather than undertaking this at the time of relapse the sequencing takes place using primary tumour tissue. They have collected 1000 samples of all of which are being sequenced. The data analysis is being funded by Sarcoma UK.

Next Generation Sequencing (NGS), at the heart of the 100,000 Genome project and the fundamental science behind precision medicine, is being welcomed by some in sarcoma research but raises concerns for others. We are still reliant on accurate histopathology and clinical decisions will always be made with an awareness of the pathological diagnosis. We now have a lot of information about mutations in sarcomas of all kinds for which there is no clinical treatment. The prospect of a targeted therapy to address mutations in myxofibrosarcoma, for example, is remote in the short to medium term. The patient group is more elderly, often with a poor performance status, not attractive to pharmaceutical companies, as well as having small numbers which are widely distributed.

##### Tissue banking and access to samples

In the days when an individual’s personal data is a sensitive issue and subject to the introduction of new regulation the issue of tissue banking takes on a particular delicacy. It is a subject close to patients. When asked if they are happy for tissue samples to be saved for research very few patients say no. But consent for donation is the easiest part of the process.

In the past tissue collections were built up during a clinical trial, but hold consent to distribute tissue to researchers which is incomplete by current standards. These tissue banks are a valuable resource but are limited because they are prevented from wider use, the very purpose for which the tissue was donated. Legislators have not fully resolved these issues yet.

New tissue donations which have modern consent and data confidentiality associated with them are now growing. There is good guidance available and there are also the tools which allow tissue banks to be ‘virtual’—standard operating procedures and protocols implemented locally for donation and storage, data held on a central database allowing researchers to identify what they wish to use, and centralised procedures for distribution (and payment) once appropriate regulatory and ethics approvals are in place.

Some kind of cross-border structure would certainly facilitate sarcoma research. Whether we can develop agreement for a single tissue bank for sarcoma operating on these principles across all countries where sarcoma MDT networks exist currently seems a remote hope. This is an area with some unusual funding issues. Currently we know of no plans to develop such a structure within the sarcoma community.

##### Follow-up and survival

We can identify that with the exclusion of GIST, sarcoma is making slower progress in improving survival than many cancers. Increasing survival puts pressure on follow-up as more patients have to be seen and tested at regular intervals. There are no simple protocols for sarcoma follow-up published in guidance from ESMO or NCCN, although the latter is firmer in making recommendations. One of the problems is that there are no generally accepted and validated risk assessment tools supported by evidence, meaning that individual clinical judgement is required.

Follow-up can be a burden for patients, although it can also offer reassurance. Follow-up is also a burden on clinical time which specialist clinicians accept but with a survival rate now extending over 50% there have to be better ways of using scarce expertise. Clinical judgement is needed to determine who is a high risk patient and requires more intensive follow-up, and who is a low-risk patient and may be seen less frequently, or perhaps can be followed in primary care or by a trained nurse. Every healthcare system is different and we lack the exchange of experience of the kind that happens with the more intensive aspects of treatment.

The point when a patient can be regarded as a survivor is also an interesting one for sarcoma patients. We know that low-grade tumours can recur late, often 8 years or more following primary treatment, and 20 years has been reported anecdotally. Higher grade tumours can also follow a more indolent pattern of intermittent recurrence. The tumour histology is an influence on the pattern of recurrence as well as the effectiveness of primary treatment. This shrouds the issue of survivorship with uncertainties for sarcoma patients. One patient has described it thus, “treatment is a process we can understand, survival is a black hole”. This also increases the follow-up challenge for clinicians. There are few publications which help us understand this tumour behaviour, and which attempt to address the specific survival needs of sarcoma patients.

### Section 5—Research

The heterogeneity of sarcoma presents researchers and research organisations with many challenges and patient advocates try and help address those challenges. However, patients have the uncanny knack of also identifying gaps. Scientists and research clinicians may know about these gaps but may regard them as beyond their capability to resolve, or see them as of lower priority than matters immediately calling for their attention. Tissue banking, discussed above, is an example which falls into both those categories.

Perhaps the most valuable role of patient advocacy groups is developing and making available information about research, especially clinical trials which are open to patients. Such trials are rarely open in every treatment centre so knowing where a trial is available is a key element in the information. Until recently an individual patient (or even a clinician) would find it difficult to get that information. SPAEN now has a trials listing which details trials which are open and provides links to trial websites for the latest data. Sarcoma UK has a similar trials ‘hub’ for studies open in the UK and the Italian Associazione Paola has one for trials in Italy.

It is our hope that the lessons which have been learned in creating these information hubs can be taken on by other patient advocacy groups in their own countries.

#### The priority challenge—patient involvement in research

One of the challenges for the patient community for many years has been patient involvement in research. The saying “nothing about us without us” is over-used but nonetheless appropriate. There is growing evidence that patients involved with research have a lot to offer, asking questions, providing reference experiences, sometimes just being there, changes the dynamics. It is important to recognise that patients probably have more to offer in research studies in care, those looking at methods and standards, and in reviews, including systematic reviews, than they do in drug related studies. Nonetheless those with particular understandings, experience or knowledge of research do offer strengths in that area too.

In far too many instances patient involvement is ad hoc. This means that it tends to be without direction, training is poor, and although a recognition of the value in having patients involved is generally expressed by researchers there is no continuity and little or no evaluation which supports the move. This means that while academic groups have more regularly engaged with patients few pharmaceutical companies or CROs have done so in a consistent and sustainable manner. This is not unique to sarcoma.

Our partnership in sarcoma research is still developing and will be for many years. The roles which patients can play need to be better defined. We also have very few patients or carers prepared or able to be involved. Involvement should be considered as an integral part of the research work—whether that is a programme of research or a single study. We can applaud research programmes which have received European Commission funding—Conticanet, EuroSarc and EuroEwing. Patients are also now attending the meetings of the EORTC Bone & Soft Tissue Sarcoma Group. Nationally only the UK and France have developed methods for patient involvement in national research programmes in sarcoma.

There is a lot of discussion on this issue in the wider world of patient involvement, particularly looking for evaluation which can tip arguments in favour of researchers engaging with patient advocates and which can point to methods and approaches which draw identifiable value out of the partnership. A recent study concluded that evaluation of patient involvement looked at it as an ‘intervention’ and has tried to assess it that way, considering outcomes. It concluded that an approach evaluating the process was more appropriate [15]. Clearly quite a lot more work needs to be done if patient input to research is to become truly effective.

One limiting factor we face in sarcoma is a direct result of the small numbers of patients we have. The language of science is English and patients wishing to be involved in research outside their own national boundaries require competence in both written and spoken English. However even in the UK finding patients with a willingness to represent patient interests in sarcoma research is proving challenging.

#### One last challenge

I want to raise one question which we can identify as patient advocates. The non-responsiveness of most sarcoma sub-types to chemotherapy, the failure of targeted therapies even when a relevant biomarker is present, the generally poor overall survival even, it seems, with immunotherapy which works well with other tumour types, all give rise to the question:

- Are we asking the right research questions?

As patients and professionals together we need to re-visit this question on a regular basis.

#### Research footnote

Research has an additional consideration for patient advocates. There is evidence emerging that hospitals which are active in research have better outcomes than those which disregard research. This appears to be an absolute factor, dependent on research active departments positively affecting the whole hospital [16]. It complements a frequent feeling among patient advocates that engaging with research active clinicians means that they are working with the best, with the doctors most likely to deliver better outcomes for the patients they support.

## Looking at international sarcoma advocacy

Sarcoma Patients Euronet now has more than 40 member associations across the world. The majority are in Europe. While individual members will usually be the most effective advocates in their own countries SPAEN acts across Europe with the pan-European institutions and is developing a programme of advocacy education and support accessible through its annual meeting and co-operative arrangements with other organisations such as WECAN, ESO and EORTC. SPAEN members also attend international meetings wherever possible.

### EU and UK

It is too easy to muddle sarcoma patient advocacy with rare cancer patient advocacy. The two groups overlap, of course, but there are sarcoma issues which are not general rare cancer issues, just as other rarer cancers have their own requirements. This means that leaving rare cancer initiatives to fully represent sarcoma, or organisations which have no patient experience of sarcoma to represent sarcoma, has the potential to miss opportunities and even cause problems. There are many initiatives aimed at the European Union institutions which SPAEN cannot be involved with for practical reasons, resources are scarce, but which could impact on sarcoma care.

SPAEN membership is not EU dependent. It has a worldwide membership.

### Eastern Europe

The big challenge with Eastern Europe is persuading healthcare systems, which are largely underfunded by the state and have little or no insurance funded structure, to establish specialist care for sarcomas. Sarcoma is not alone with this issue and European initiatives may help but the European Union has no powers over national healthcare systems. The SPAEN Policy Paper, already mentioned, and our collaboration on developing publications in partnership with our specialists, are hopefully starting to help address this challenge by bringing a comparison with full and proper care to the notice of healthcare authorities.

### Rest of the world

SPAEN’s involvement with organisations in the rest of the world is at an early stage in development. We have member groups in India, Israel, Turkey and the USA, some for some years, especially with regard to GIST. The US groups are well established. Some of them pre-date SPAEN. Others are less well established. Developing this worldwide interest effectively is a challenge for SPAEN.

### Conflicts of interest

The funding of patient advocacy groups gives rise to consideration of conflicts of interest. The source of the most significant funding for patient groups is industry, and most specifically the pharmaceutical industry. Some countries do provide government grants or allowances, although these are small, and voluntary donations are a further important source for many groups. Running a formal membership roll with subscriptions creates an administrative burden which is an added cost which some are not prepared to bear. Accepting support grants from industry, with an agreed and carefully managed structure to avoid conflicts, is a step which any patient organisation seeking to create consistent impact needs to do, even if it raises concern among watchers.

The important issue is openness. Arrangements must be transparent, it should be clearly stated that funding carries no right to control the public stance of the patient organisation in any aspect of its work. Ideally the patient organisation will have a range of funding agreements so it is not dependent on only one financial supporter, and the nominated use of any specific funding should not relate to the products or activities of the funder. Some funders are happier supporting projects rather than the underlying costs of a group. While this can be respected groups do need to have ‘core’ funding to exist at all.

Openness also means that in contacts with regulatory bodies the patient organisation should be open about its funding and its funders. A recent analysis of attendees at an FDA ODAC meeting [17] showed that 30% of the patient group representatives had a potential for conflict, but this was lower than most other groups attending. EMA in particular is quite rigorous about declaring any potential for conflict, prior to a meeting and during a meeting if relevant.

## Conclusion

The danger that this review could look like a ‘shopping list’ is one I have been conscious of from the very start. It would also be easy for it to look negative, as if nothing is happening and there are few plans, but the truth is that things are moving along in a positive direction although there is a long journey yet to travel. It has taken a lot of hard work to start addressing these challenges, involving a substantial number of people, many clinical professionals and sarcoma health specialists, patients and patient advocates, regulatory and healthcare influencers, both political and administrative.

As a community however we must be able to find ways of creating beneficial change without needing to resort to the excuses of ‘heterogeneity’ or ‘rarity’. These two factors are inescapable truths, part of our DNA so to speak. We must learn to accommodate them and move forward.

I will highlight once more as requiring action with some immediacy the following challenges:

- Earlier accurate diagnosis and primary treatment
- Multi-Disciplinary Management
- Cancer registration and patient data
- Quality of life and PROs
- Patient involvement

Two of the other challenges only just missed out on being priorities when I was identifying the leading challenge in each Section.

- Second opinion, referral and reference networks
- Innovation in clinical trials and drug regulation

Above all there is little doubt from all the evidence I have seen, and having questioned clinical specialists of many nationalities around the world, that the greatest impact on long-term disease-free survival, also known as cure, will come from earlier, accurate diagnosis. Larger tumours are indicative of a poorer outcome, it is that simple.

## The author

The author, Roger Wilson, was diagnosed with a soft tissue sarcoma in 1999. He has faced six recurrences, ten surgical operations, radiotherapy and chemotherapy. His treatment has included an amputation, two thoracic metastectomies, and treatment on an EORTC clinical trial. He founded Sarcoma UK in 2003 and was a co-founder of Sarcoma Patients Euronet in 2009, serving on its Board since then. He is currently Honorary President. He has worked as a patient advocate in both sarcoma and cancer research since 2002, serving on the Board of the UK’s National Cancer Research Institute (NCRI) for 4 years. He is a member of the NCRI Sarcoma Clinical Studies Group. In Europe he has worked with EORTC, ESMO and ECCO.

## Data Availability

All material has been referenced except certain unpublished public data recorded in conference presentations. This has been noted in the text. No data was originated in this study.

## Abbreviations

A&E: accident and emergency department
ACS: American Cancer Society
CNS: clinical nurse specialist
EMA: European Medicines Agency
ESMO: European Society for Medical Oncology
ESO: European School of Oncology
EORTC: European Organisation for Research and Treatment of Cancer
EURACAN: European Rare Cancer Reference Network
GIST: gastro-intestinal stromal tumour
MDT: Multi-Disciplinary Team
NGS: next generation sequencing
NCCN: National Comprehensive Cancer Network
NHS: National Health Service (UK)
PROs: patient reported outcomes
RPS: retroperitoneal sarcomas
QoL: quality of life
SPAEN: sarcoma patients euronet
WECAN: workgroup of European cancer patient advocacy networks
